# Cost-effectiveness of single-use negative-pressure therapy compared with standard care for prevention of reconstruction failure in prepectoral breast reconstruction

**DOI:** 10.1093/bjsopen/zraa042

**Published:** 2021-04-11

**Authors:** J A Murphy, D Myers, P Trueman, R Searle

**Affiliations:** Nightingale Breast Unit, Manchester University NHS Foundation Trusts, Manchester, UK; Smith and Nephew, Hull, UK; Smith and Nephew, Hull, UK

## Abstract

**Background:**

Single-use negative-pressure wound therapy (sNPWT) has been reported to reduce the incidence of reconstruction failure in prepectoral breast reconstruction compared with standard surgical dressings. The aim of this economic evaluation was to investigate the cost-effectiveness of sNPWT compared with standard care for the prevention of reconstruction failure in prepectoral breast reconstruction in the UK.

**Method:**

A decision tree model was used to estimate the expected cost and effectiveness per patient. Effectiveness was measured both by the number of reconstruction failures avoided and the gain in quality-adjusted life-years (QALYs). The baseline incidence of reconstruction failure (8.6 per cent) was taken from a recently published study of 2655 mastectomies in the UK. The effectiveness of sNPWT used results from a clinical study comparing sNPWT with standard dressings. Previously published utility weights were applied. The cost of reconstruction failure was estimated from detailed resource data from patients with reconstruction failure, applying National Health Service reference costs. One-way, probabilistic, scenario and threshold analyses were conducted.

**Results:**

The undiscounted cost per patient associated with reconstruction failure was estimated to be £23 628 (£22 431 discounted). The use of sNPWT was associated with an expected cost saving of £1706 per patient, an expected increase in QALYs of 0.0187 and an expected 0.0834 reconstruction failures avoided. Cost-effectiveness acceptability analysis demonstrated that, at a threshold of £20 000 per QALY, 99.94 per cent of the simulations showed sNPWT to be more cost-effective than standard care.

**Conclusion:**

Among patients undergoing immediate prepectoral breast reconstruction, the use of sNPWT is more cost-effective than standard dressings.

## Teaser

The aim of this economic evaluation was to investigate the cost-effectiveness of single-use negative-pressure wound therapy (sNPWT) (PICO™) compared with standard care for the prevention of reconstruction failure in prepectoral breast reconstruction in the UK. Using a decision-analytical model, the use of sNPWT was associated with an expected cost saving of approximately €1880 per patient, an expected increase in quality-adjusted life-years of 0.0187, and an expected 0.0834 reconstruction failures avoided. This analysis suggests that use of sNPWT is more cost-effective than standard dressings for women undergoing immediate prepectoral breast reconstruction.

## Introduction

Breast cancer is the most common cancer in adult women, with around 55 000 new cases every year in the UK^1^^,^^2^. Over the past 25 years, incidence rates have increased by around 20 per cent, and continue to rise^1^^,^^2^. Survival has, however, improved, with over two-thirds of women surviving for at least 20 years from diagnosis^1^. The majority undergo either breast-conserving surgery (around 60 per cent) or mastectomy (40 per cent), the latter often being the procedure of choice if the shape of the breast cannot be preserved^3^^,^^4^. Between 2013 and 2015 there were 111 000 breast cancer diagnoses in England, with 81 per cent of patients undergoing cancer resection surgery in the 12 months after diagnosis^5^. When mastectomy is chosen, immediate reconstruction is offered when appropriate, with around 40 per cent of these patients undergoing such procedures^6^. Immediate implant-based reconstruction (IBR) is used widely, offering good cosmetic outcomes and a relatively short recovery time^6^^,^^7^. A subset of IBR, known as prepectoral reconstruction, is a relatively recent development in which the implant is positioned in front of the pectoralis muscles. This approach has been associated with quicker recovery, less donor site morbidity, and fewer functional problems compared with implants placed behind the pectoralis muscle^8^

A significant complication of prepectoral reconstruction is impaired wound healing, which may lead to infection, reconstruction failure and, potentially, loss of the implant^8–10^. A multicentre UK study^11^ found an implant loss rate of around 9 per cent at 3 months. Reconstruction failure may be associated with increased resource utilization for the health system, including hospital attendances and readmissions^12–14^. Further surgical procedures, such as explantation, insertion of tissue expanders and reimplantation, may be necessary^12^.

Single-use negative-pressure therapy wound therapy (sNPWT) has been shown to be effective in the prevention of surgical incision complication rates^15^^,^^16^. A systematic review and meta-analysis^15^ of 1863 patients across multiple specialties demonstrated an average reduction in the surgical-site infection rate of 58 per cent (from 12.5 per cent to 5.2 per cent). In breast surgery, a comparison of sNPWT with standard care found that sNPWT was associated with a significant reduction in the incidence of wound complications and dehiscence^17^. Following mastectomy and implant-based reconstruction, the use of sNPWT led to a reduction in the incidence of reconstruction failure compared with standard surgical dressings. The cost of a reconstruction failure was estimated to be over £14 000, taking into account the need for multiple readmissions to hospital and outpatient visits^12^.

A more formal structured economic evaluation, using cost-effectiveness analysis based on decision-analytical principles, would help policy-makers to decide whether the use of sNPWT is sensible. This type of analysis evaluates the balance between additional clinical benefits obtained from an alternative treatment and its incremental cost. Although the cost-effectiveness of various procedures, techniques and products used in breast reconstruction surgery and the economic benefits of sNPWT have been reported, the cost-effectiveness of sNPWT for the prevention of breast implant loss has not been investigated^11^^,^^13^^,^^18^^,^^19^. The aim of this economic evaluation was therefore to investigate the cost-effectiveness of sNPWT using PICO™ (Smith and Nephew, Hull, UK) compared with standard care for the prevention of reconstruction failure in IBR.

## Methods

### Decision-analytical model

**Fig. 1:**
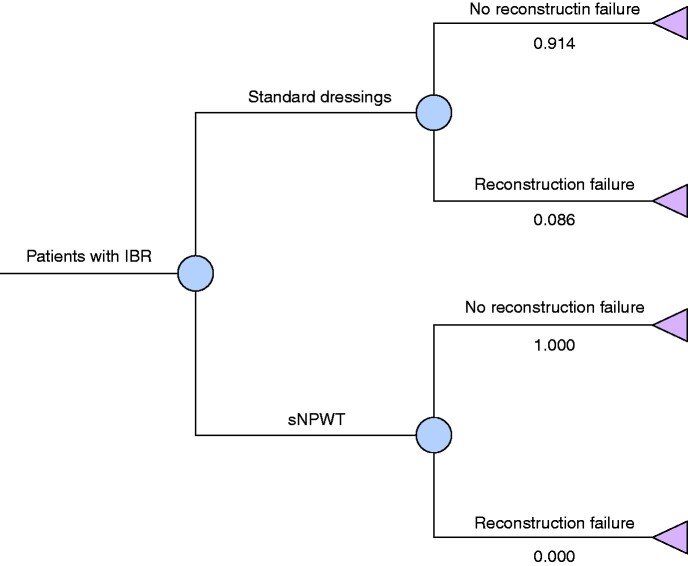
Decision-analytical model Squares are decision nodes and circles are chance nodes. The numbers following a chance node refer to conditional probabilities. The probabilities for all branches from a chance node add to 1. For example, for single-use negative-pressure wound therapy (sNPWT), no patients have reconstruction failure. IBR, immediate implant-based reconstruction.

The evaluation was designed to compare the expected incremental cost and clinical consequences for a cohort of women treated with standard care (surgical dressings including transparent waterproof dressings with an absorbent pad) and a cohort treated by sNPWT. A decision tree model was constructed (*Fig. 1*).

In each arm of the model, patients can have either reconstruction failure or no failure. The analysis was conducted from the perspective of the UK health system; the time horizon was 48 months to reflect the consequential cost of reconstruction failure. Two measures of effectiveness were considered: the number of reconstruction failures avoided and the number of quality-adjusted life-years (QALYs) gained.

A study^11^ of 2655 mastectomies in 2108 women undergoing IBR at 81 UK centres demonstrated that 8.6 per cent of patients experienced implant loss at 3 months. This rate was used as the baseline incidence of reconstruction failure in the decision-analytical model, and is similar to the rate reported previously in a National Mastectomy and Breast Reconstruction Audit of 8.9 per cent^3^. A study published in 2020 comparing sNPWT with standard dressings reported no reconstruction failures in the sNPWT group, compared with 4.3 per cent in the control group.^13^ This relative risk was used as the base case and explored further in sensitivity analyses. The same study^13^ was used to make assumptions regarding the proportion of patients undergoing bilateral surgery (307 reconstructions in 196 patients).

### Resource utilization and unit costs

To estimate the resource use associated with reconstruction failure, deidentified data were obtained in from the study by Irwin and colleagues13, which detailed postsurgical outpatient visits and admissions for five patients with seven reconstruction failures from a total of 181 breast reconstructions. To ensure that these resources reflected those associated with reconstruction failure, the normal pattern of resource use for postsurgical recovery without complications was subtracted. This was assumed to be three outpatient visits for each patient: one for dressing change and two for consultant review. After adjusting for the normal pattern of resource usage, this was modelled as 21 admissions subsequent to the original procedures, 57 additional outpatient visits for dressing change, and 83 additional consultant-led outpatient visits.

To convert resource utilization to costs an appropriate Healthcare Resource Group code was assigned to each item, with each level of resource multiplied by the National Health Service (NHS) 2018–2019 reference cost (*Table 1*)^20^. Costs were not assigned to emergency department visits as they all resulted in admission, and the assigned emergency admission cost was assumed to include such costs. The cost of two acellular dermal matrices per breast reconstruction (£4020) was added, as these represent an additional cost to the health system. The unit cost of the PICO™ NPWT system (£147.06) was obtained from NHS Supply Chain, with averaging of the unit cost across the various sizes available and in the base case assuming that one NPWT system was used per incision. As a conservative approach, the relatively low cost of standard dressings was not included in the model.

**Table 1:** Assignment of unit costs to resources associated with reconstruction failure

Resource category	Healthcare Resource Group code	**Unit cost** **(2018–2019 reference costs)**
**Hospital admissions**		
Implant removal	JA43B: unilateral intermediate breast procedures with CC score 0–2	£1167
	JA42Z: bilateral intermediate breast procedures	£1662
Insertion of tissue expanders, exchange of implants, tissue lipomodelling and tissue expander insertion or inflation	JA20F: unilateral major breast procedures with CC score 0–2	£2542
	JA21B: b ilateral major breast procedures with CC score 0	£3581
Emergency admission with antibiotics and seroma drainage	WH07G: infections or other complications of procedures, without interventions, with CC score 0–1	£2056
**Outpatients**		
Dressing change visit	WF01A: follow-up attendance, single professional	£130
Consultant-led review visits	WF02A: follow-up attendance, multiprofessional	£164

Where appropriate the cost of two acellular dermal matrices was added, as these represent an additional cost to the health system. CC, complexity and co-morbidity.

### Health state utility values and calculation of QALYs

Cost-utility analysis measures health outcomes as a combination of life duration and health-related quality of life over that duration. The most commonly used measure is the QALY. One year of perfect health corresponds to 1 QALY, and 0 QALYs are attributed to death. One year of being in a compromised health state assigned with a utility value 0.5 would correspond to 0.5 QALYs, and so on. Health state utility values were therefore required for the two health states: reconstruction failure and no reconstruction failure. These values were used to generate QALYs by multiplying by the length of time spent in each health state. Previous publications^21^^,^^22^ have reviewed utility values in breast cancer and surgery, and provided values for an economic evaluation of the effectiveness of acellular dermal matrix in expander–implant immediate breast reconstruction^23^. A utility of 0.585 corresponded to the explantation health state, and a value of 0.70 corresponded to successful surgery^24^.

**Fig. 2:**
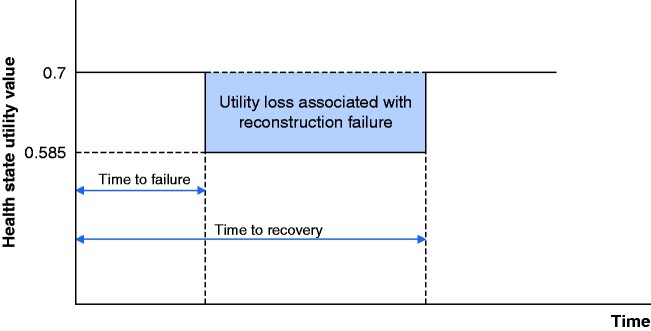
Calculation of quality-adjusted life-years

To calculate the number of QALYs associated with reconstruction failure, it was assumed that patients spent an initial period in which surgery was successful (at utility value 0.70), followed by a period at a reduced utility of 0.585, and a return to successful surgery up to the end of the time horizon (*Fig. 2*). In the case of no reconstruction failure, a value of 0.70 was assumed from immediately after surgery until the end of the time horizon. As utility values vary depending on the source, the exact definition of the health state and the method used to elicit the data, variations in these values were explored in the sensitivity analysis.

### Sensitivity analysis

Sensitivity analyses were used to identify which model inputs had the greatest effects on the model results, as well as testing the model under different conditions, to check the robustness of the conclusions. Several sensitivity analyses were undertaken. A one-way sensitivity analysis was performed in which each model input was varied in turn by ±20 per cent, leaving all other inputs unchanged, and the results were plotted in order of greatest effect on the incremental cost. This was followed by a series of scenario analyses, using lower and higher discount rates (0 and 6 per cent), lower utility of successful surgery (0.6), higher utility of reconstruction failure (0.6), two sNPWT systems per breast reconstruction instead of one, lower baseline reconstruction failure rate of 4.33 per cent (estimated from Irwin *et al*.^13^), and reduced cost of reimplantation.

To investigate uncertainty in the model parameters, a probabilistic sensitivity analysis (PSA) was undertaken. This was an iterative process in which the model was run a large number of times. For each iteration, the value of each model parameter was set by drawing a value from a predetermined distribution. The type of distribution and its parameters were determined from the type of model input, its point value, and a measure of its variability *(Table 2*). The risk of failure for the sNPWT cohort presents a problem for PSA, because Irwin and colleagues^13^ observed no reconstruction failures in this group, so the base-case relative risk was zero. When α was set as 1, after setting β to result in a mean value of 0.01, this gave a value for β of 115.56.

**Table 2:** Model input distributions and parameters used in the probabilistic sensitivity analysis

Model input	Distribution	Parameters
Baseline risk of reconstruction failure with standard care	β	α = 182 β = 1899
Risk of failure for sNPWT	β	α = 1^*^ β = 115.56^*^
Cost per patient associated with reconstruction failure	γ	α = 90.62 β = 260.74
No. of sNPWT systems	Fixed	1 per breast reconstruction (average of 1.566 per patient)
Disutility – reconstruction failure	γ	α = 25 β = 0.0166
Disutility – no reconstruction failure	γ	α = 25 β = 0.012
Time taken for failure to occur	γ	α = 35.266 β = 0.032
Time elapsed before failure was resolved	γ	α = 36.639 β = 0.683

*α was set at 1 and then β was set to 115.56, to result in a mean value of 0.01. sNPWT, single-use negative-pressure wound therapy.

The variability of utility weights was unavailable, so a standard error of 20 per cent of the mean value was assumed. As the number of QALYs associated with reconstruction failure also depended on the duration of the period at reduced utility, the time taken for failure to occur and the time that elapsed before the failure was resolved were also varied. Although the cost of reconstruction failure and the utility associated with failure might be expected to be correlated, a lack of data to inform the strength of this relationship meant that these variables were not considered in the PSA model. Once the incremental cost and effectiveness had been calculated for each simulation, the net monetary benefit was calculated for each pair of values at a series of different willingness-to-pay thresholds. These were then used to determine the proportion of simulations where sNPWT was more cost-effective than standard dressings^25^.

Three threshold analyses were conducted, in which the cost of reconstruction failure, the baseline risk of reconstruction failure, and the risk of reconstruction failure using sNPWT were changed until the incremental cost became cost-neutral with respect to standard dressings.

### Discounting

Discounting accounts for differences in the time at which costs and benefits occur, present costs and benefits having a higher value than future costs. Because the time horizon exceeded 1 year, costs and effects were both discounted at an annual rate of 3.5 per cent^16^. To apply a discount rate to the cost of reconstruction failure, resource data were used to estimate the cost incurred in years 1–4, and then discounted using these values. In the PSA, each iteration generated a different total cost of reconstruction failure. To discount these values it was assumed that the proportion of cost incurred in each year was the same as in the base case. QALYs were discounted using the times to failure and recovery (*Fig. 2*). For the discounting of the number of reconstruction failures, it was assumed that the failures occurred in year 1 and were discounted accordingly.

## Results

### Cost associated with reconstruction failure

The undiscounted cost per patient associated with reconstruction failure was estimated to be £23 628 (£22 431 discounted).

### Base case analysis

On a per-patient basis, the base case analysis demonstrated that the use of sNPWT was associated with an expected cost saving of £1706.29, an expected increase in QALYs of 0.0187, and an expected 0.0834 reconstruction failures avoided (*Table 3*).

**Table 3:** Base case results

	Expected value (per patient)		
	**Standard dressings**	**sNPWT**	**Incremental**
Proportion of patients experiencing reconstruction failure	0.0834	0	−0.0834
QALYs	2.5524	2.5711	0.0187
Cost (£)	1936.63	230.34	−1706.29

Due to rounding, some totals may not correspond with the sum of the separate values. sNPWT, single-use negative-pressure wound therapy; QALY, quality-adjusted life-year.

### One-way sensitivity analysis

**Fig. 3:**
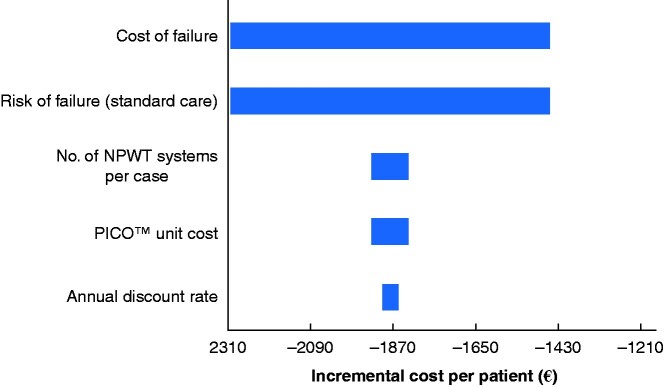
Tornado diagram showing the results of the one-way sensitivity analysis NPWT, negative-pressure wound therapy;

The results of the one-way sensitivity analysis are shown in the Tornado diagram in *Fig. 3*. This shows, for each model input, the range of values of incremental cost generated by the one-way analysis. The extremes of each range represent the results for the base case value ±20 per cent. The results are presented in decreasing order of the magnitude of the range. Therefore, the variables are shown in decreasing order of their impact on the incremental cost. The cost of reconstruction failure and risk of failure had the greatest impact on incremental cost. Three associated threshold analyses were undertaken.

### Probabilistic sensitivity analysis

**Fig. 4:**
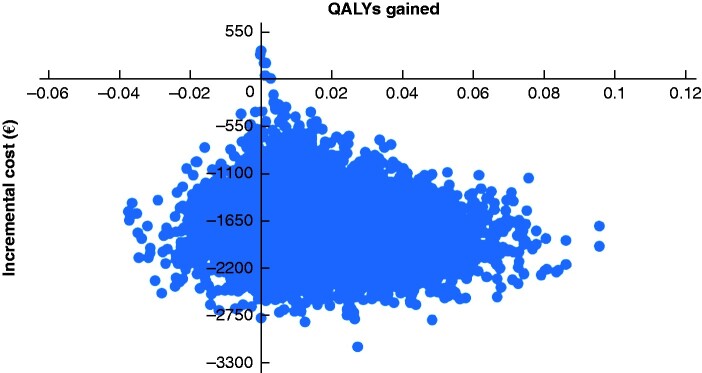
Results of the cost-utility analysis The distribution is shown of 10 000 pairs of incremental cost and incremental quality-adjusted life-years (QALYs) per patient, obtained from the probabilistic sensitivity analysis. The majority of the points lie in the south-east quadrant, indicating that single-use negative-pressure wound therapy is dominant (more effective and less costly) over standard dressings.

**Fig. 5:**
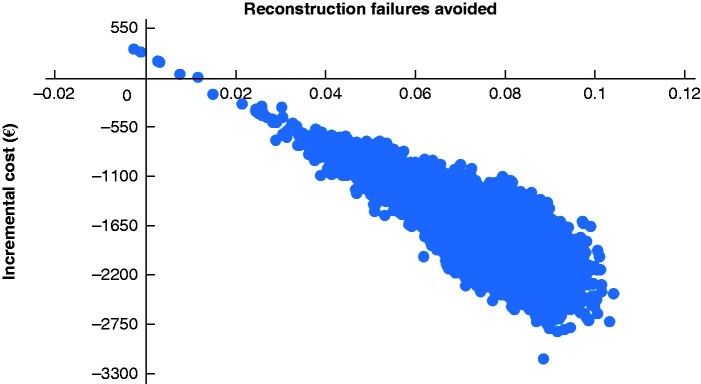
Results of the cost-effectiveness analysis The distribution is shown of 10 000 pairs of incremental cost and incremental reconstruction failure per patient obtained from the probabilistic sensitivity analysis. The majority of the points lie in the southeast quadrant, indicating that sNPWT is dominant (more effective and less costly) than standard dressings.

After 10 000 simulations, the probabilistic analysis resulted in dominance of sNPWT over standard care, with an expected cost saving of £1539 per patient, 0.076 reconstruction failures avoided per patient, and 0.0170 QALYs gained per patient. Where ‘failures avoided’ was the effectiveness measure, 99.94 per cent of the 10 000 simulations resulted in dominance of sNPWT over standard care. For health-related quality of life (cost-utility analysis), sNPWT dominated in 86.79 per cent of simulations. *Figs 4* and *5* show the distribution of 10 000 pairs of incremental cost and incremental QALYs per patient obtained from the probabilistic sensitivity analysis. The majority of the points lie in the south-east quadrant, indicating that sNPWT is dominant (more effective and less costly) over standard dressings. A relatively small number of simulations fall outside the south-east quadrant, indicating that the uncertainty in the result—that sNPWT dominates standard dressings—is small.

Cost-effectiveness acceptability analysis estimated the proportion of simulations in which sNPWT was more cost-effective than standard care at a range of willingness-to-pay thresholds. These thresholds are a measure of the cost per QALY gained that the health system is willing to pay. At the threshold recommended by the UK National Institute for Health and Care Excellence (NICE) of €22 000 (£20000), 99.94 per cent of the simulations showed sNPWT to be more cost-effective than standard care.

### Scenario analysis

Results of the scenario analyses are shown in *Table 4*.

**Table 4:** Results of the scenario analyses

Scenario	Expected incremental cost per patient (£)	Expected reconstruction failures avoided per patient	Expected QALYs gained per patient	**ICER** ^∗^ **(€/QALY)**
Undiscounted costs and effects	−1809.63	0.0863	0.0198	Dominant
Costs and effects discounted at 6 per cent	−1638.03	0.0815	0.0180	Dominant
Lower utility of successful surgery (0.6)	−1706.29	0.0834	0.0024	Dominant
Higher utility of reconstruction failure (0.6)	−1706.29	0.0834	0.0163	Dominant
Patients require two sNPWT systems per breast reconstruction rather than one	−1475.94	0.0834	0.0187	Dominant
Baseline reconstruction failure rate of 4.33 per cent (estimated from Irwin *et al*.^13^)^†^	−740.91	0.0418	0.0094	Dominant
Reduced cost of reimplantation using reference costs only; total cost of reconstruction failure £13 980 per patient	−921.88	0.0834	0.0187	Dominant

*The description ‘dominant’ indicates that single-use negative-pressure wound therapy (sNPWT) is expected to be both cost-saving and more effective than standard dressings.

^†^The rate of reconstruction failure per patient was estimated using the proportion of reconstruction failures per breast and the proportion of bilateral incisions. QALY, quality-adjusted life-year; ICER, incremental cost-effectiveness ratio.

### Threshold analysis

The per-patient cost incurred as a result of reconstruction failure was reduced (all other model inputs remaining the same) until, at an undiscounted cost of £2810 (discounted cost of £2668), the incremental cost became zero. Therefore, if the cost of reconstruction failure was above this point, the use of sNPWT would be expected to be cost-saving compared with standard care, and if below this point, it would be expected to be cost-additive. In a similar way, reducing the baseline risk of reconstruction failure suggested that above a level of 1.03 per cent the use of sNPWT is also expected to be cost-saving. In the final threshold analysis, cost-neutrality occurred when the risk of reconstruction failure with sNPWT was 7.607 per cent (a relative risk of 0.881). This means that if sNPWT can reduce the risk of failure from the assumed baseline value of 8.6 per cent to 7.6 per cent or less, sNPWT remains more cost-effective than standard dressings.

## Discussion

In England, between 2007 and 2014, immediate reconstruction rose from 30 to 54 per cent as a proportion of all breast reconstruction procedures^24^. Serious complications such as implant loss requiring explantation and reimplantation can lead to substantial additional costs. The present analysis found that the average additional cost per patient with reconstruction failure was around £23 000. The analysis suggests that sNPWT may be more cost-effective than standard dressings, resulting in expected cost savings of £1706 per patient, an increase of 0.0187 QALYs per patient and a reduction in failure rate from 8.3 per cent to zero. The small difference in QALYs is to be expected as any impairment in quality of life as a result of treatment failure is short term and assumed to be resolved by revisional surgery.

Cost-effectiveness and cost-utility analyses are widely used and accepted as tools to inform policy decision-making in healthcare, and methods are standardized through the use of guidelines^16^. The base case risk of reconstruction failure (8.6 per cent) was drawn from a large study across many centres in the UK^11^. The target rate according to breast reconstruction guidelines in the UK^26^ is less than 5 per cent, and the recent study^12^ from which the effectiveness of sNPWT was taken recorded a rate of around 4 per cent. Sensitivity analyses were therefore included based on these rates in addition to one-way analyses and a probabilistic model. All analysis techniques resulted in sNPWT dominating standard dressings.

It should be noted that, because the study compared sNPWT with standard dressings, the manufacturers of the sNPWT device have refined the product to include a more efficient, quieter pump and a ‘dressing full’ change indicator to help optimize dressing change frequency (PICO™ 7 and PICO™ 7Y). In addition, a version of the technology is available that enables the use of one pump with two dressings. Because the cost of this device is less than the cost of two sNPWT devices, its use for bilateral incisions should reduce the acquisition costs further. Although the clinical effectiveness of this new device has not been demonstrated, it is expected to be equivalent to that of the normal sNPWT device; if this were to be the case, there would be further cost savings.

This evaluation has limitations. The effectiveness of sNPWT was based on a comparative study^12^ that was not randomized. The authors^12^ concluded that sNPWT should be considered at the surgeon’s discretion. The present analysis models the cost implications of this decision, but is based on this particular study. Randomized studies would add useful information and enable the economic evaluation to be refined further. Although the cost of reconstruction failure was based on real resource data rather than assumptions, only a small number of patients were involved and data from a larger, more representative, sample would be helpful. Despite this, the sensitivity analysis indicated that the conclusions of the present economic evaluation were quite robust to changes in these costs, with sNPWT remaining the dominant treatment option.

This analysis suggests that, for patients undergoing immediate prepectoral reconstruction, the use of sNPWT is more cost-effective than standard dressings, and should be considered as a method for reducing the risk and economic impact of reconstruction failure.
